# Dataset of the catamaran floater in towing tank test with fix moment reference point

**DOI:** 10.1016/j.dib.2023.109805

**Published:** 2023-11-14

**Authors:** Sayuti Syamsuar, Karyawan, Hendrato, Yudiawan Fajar Kusuma, Muhammad, Sulistiya, Annissa Roschyntawati, Daif Rahuna, Widyawasta, Baharuddin Ali, Sutiyo, Bagiyo Suwasono

**Affiliations:** a Research Center for Transportations Technology, National Research and Innovation Agency, KST B.J Habibie, Setu, South Tangerang, Banten 15314, Indonesia; b Aerodynamics, Aeroelastic & Aeroacoustics Laboratory, National Research and Innovation Agency, KST B.J Habibie, Setu, South Tangerang, Banten 15314, Indonesia; c Research Center for Hydrodynamics Technology, National Research and Innovation Agency, KS Said Djauharsjah Jenie, Sukolilo, Surabaya, East Java 60112, Indonesia; d Research Center for Aviation Technology, National Research and Innovation Agency, KST B.J Habibie, Setu, South Tangerang, Banten 15314, Indonesia; e Hydrodynamics Laboratory, National Research and Innovation Agency, KS Said Djauharsjah Jenie, Sukolilo, Surabaya, East Java 60112, Indonesia; f Department of Naval Architecture, Hang Tuah University, Sukolilo, Surabaya, East Java 60111, Indonesia; g Postgraduate Program of Marine Coastal Engineering, Hang Tuah University, Sukolilo, Surabaya, East Java 60111, Indonesia; h Doctorate Student, Department of Mechanical, Diponegoro University, Semarang, Indonesia

**Keywords:** Catamaran floater, Trim, Speed, Force, Moment, Towing test

## Abstract

Most seaplanes used for aircraft operations in territorial waters are classified into three main types, namely floatplanes, flying boats, and amphibians. Among these, the floatplane stands out as it replaced its landing gear with two floating pontoons, known as the catamaran floater, on which the fuselage rests. Therefore, this research presented a data article on resistance testing to predict force and moment along the x, y, and z axes for a 1:10 scale model of the catamaran floater. Test data encompassed variations in trim angles 0°, −1°, and −2°, and speed ranging from 1 to 6 m/s. The results of the resistance testing are presented in the form of descriptive statistics and shown through two graphs. The first graph described the relationship between the catamaran floater's speed and the corresponding force generated. The second graph illustrated the correlation between the catamaran floater's speed and moment generated.

Specifications Table

**Table:** 

Subject	Hydrodynamics for Resistance
Specific subject area	Force (*x, y, z*) and Moment (*x, y, z*)
Data format	Design Floater and Cremona (dwg format), Towing Tank Video (mpeg format), Statistic Descriptive (xls format)
Type of data	Image/Video, Table, and Graph
Data collection	Towing tank tested floater catamaran model with a fixed movement reference point at various trim angles and speed. Test data obtained from measurements of force (Fx, Fy, and Fz) and moment (Mx, My, and Mz)
Data source location	Indonesian Hydrodynamics Laboratory - National Research and Innovation Agency, *KS Said Djauharsjah Jenie*, Keputih Village, Sukolilo District, Surabaya City, East Java 60112, Indonesia
Data accessibility	Repository name: Mendeley Data *Descriptive Floater98 with Fix-MRP in Towing Tank* DOI:10.17632/h3kmhp6zk3.2 Direct URL to data: https://data.mendeley.com/datasets/h3kmhp6zk3/2
Related research article	S. Syamsuar, Erwandi, B. Suwasono, Sutiyo, N. Kartika, A. Suksmono, A. Roshyntawati, Hendrato, AM. Kadir, A. Setyawan, A. Munazid, Numerical simulation for floater design on the 17 passenger's capacity of the N219 amphibian in static and dynamic condition, in AIP Conf. Proc. 050118 (2023) 1–8. https://doi.org/10.1063/5.0132289

## 1 Value of the Data

• The variation data, such as trim angle and speed collected from this towing tank test, provided valuable insights into the performance of force and moment generated. This is in addition to the phenomenon of water spray generated from the catamaran floater model.
• The data show the transition phase of the catamaran floater model from displacement to pre-planning or planning with a fixed moment reference point and static floating condition.
• The data is used to improve the overall understanding of the aerodynamics and hydrodynamics states of floatplane types in different operating conditions.
• The data can be used as a reference for new experiments on the catamaran floater model with an unfixed moment reference point and free-floating condition.

## 2 Data Description

**Fig. 1:**
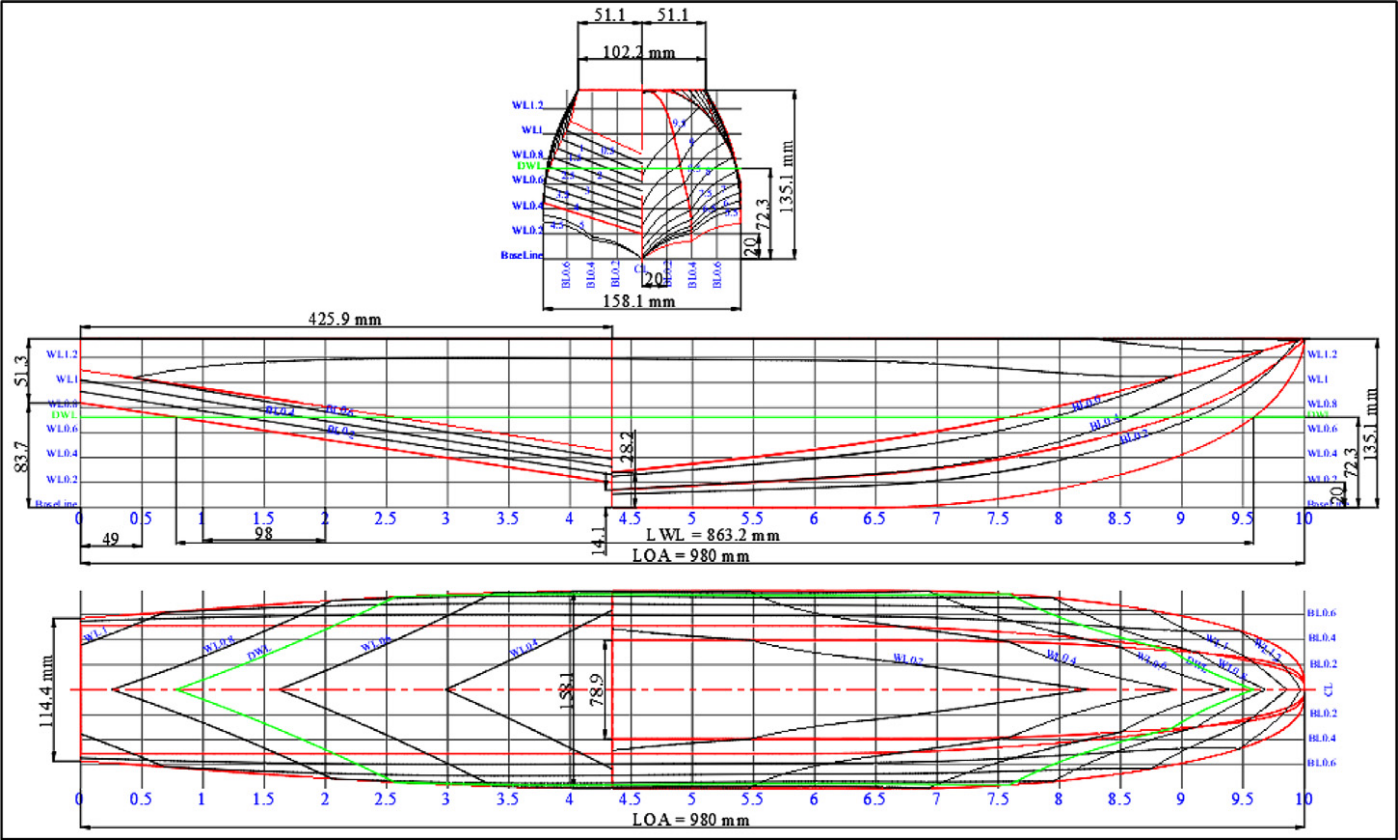
Lines plan of floater model scale 1:10.

**Fig. 2:**
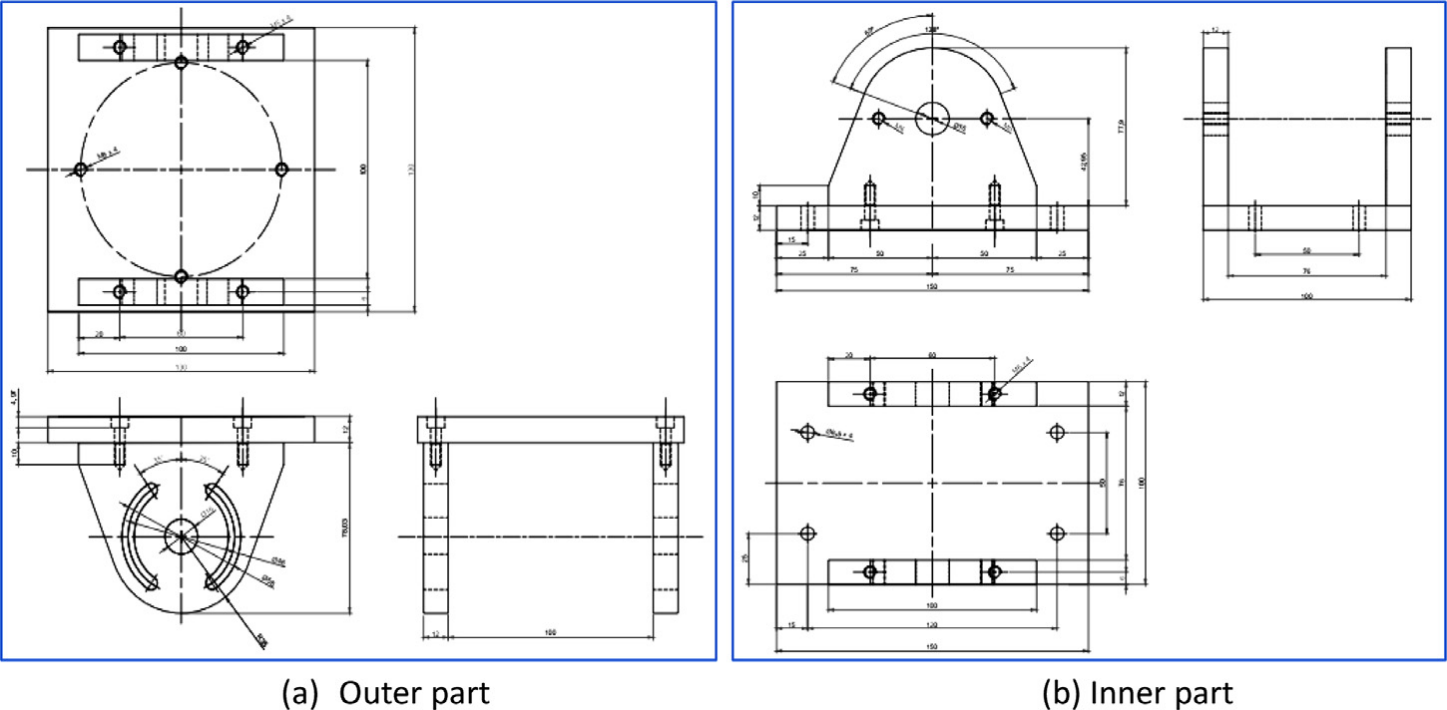
Design of fixed moment reference point.

**Fig. 3:**
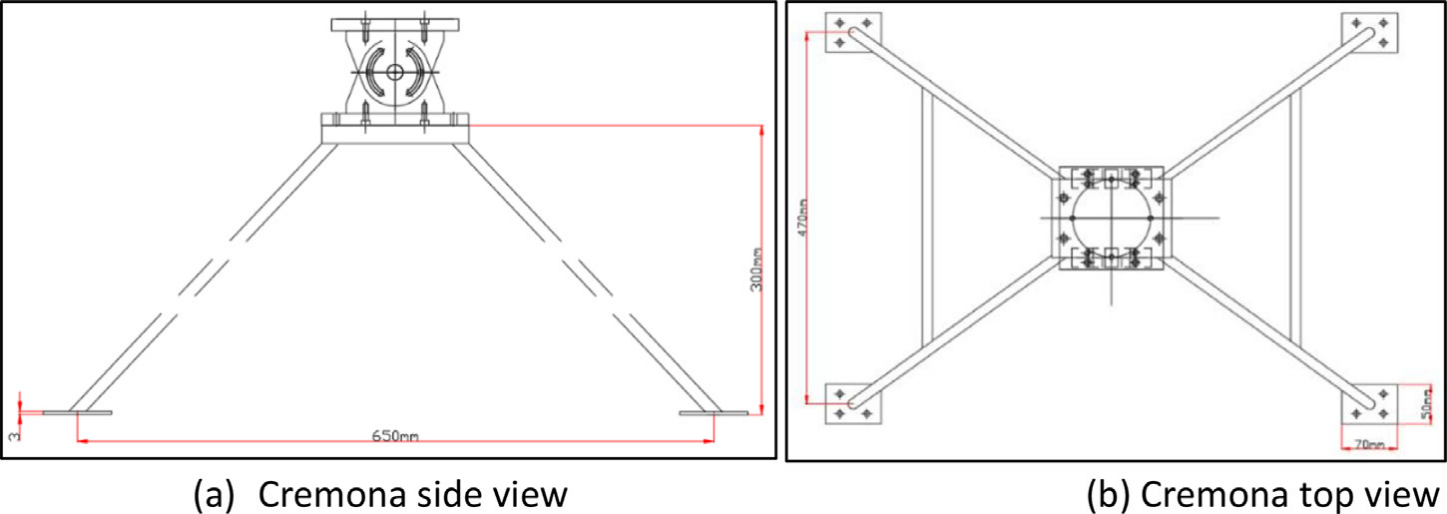
Design of Cremona.

**Fig. 4:**
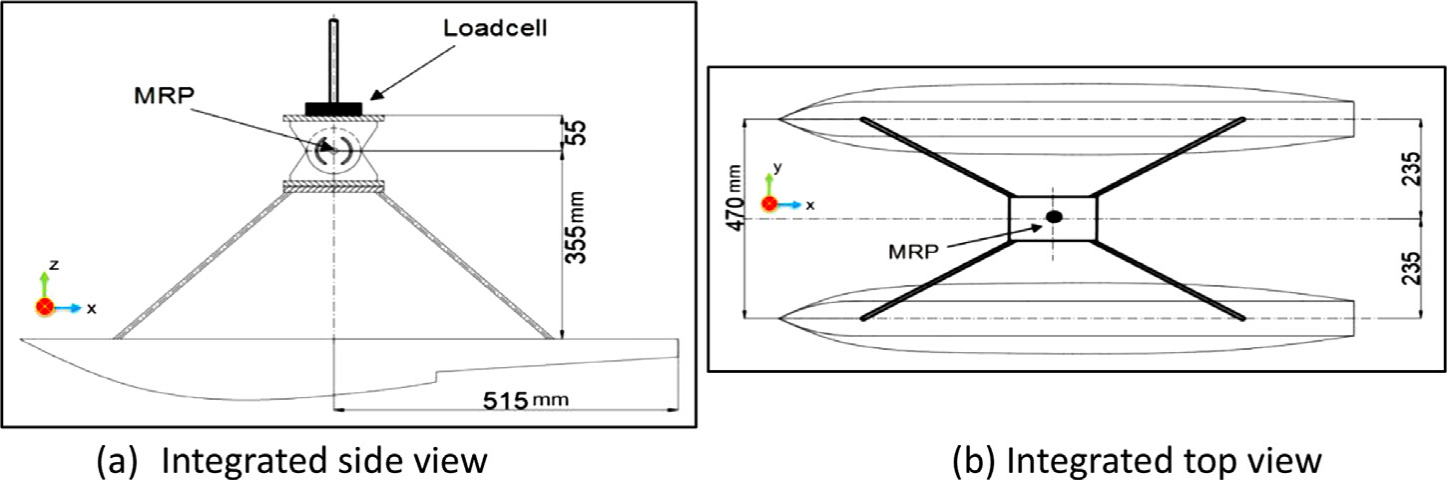
Integrated design of floater model scale 1: 10 and Cremona.

**Fig. 5:**
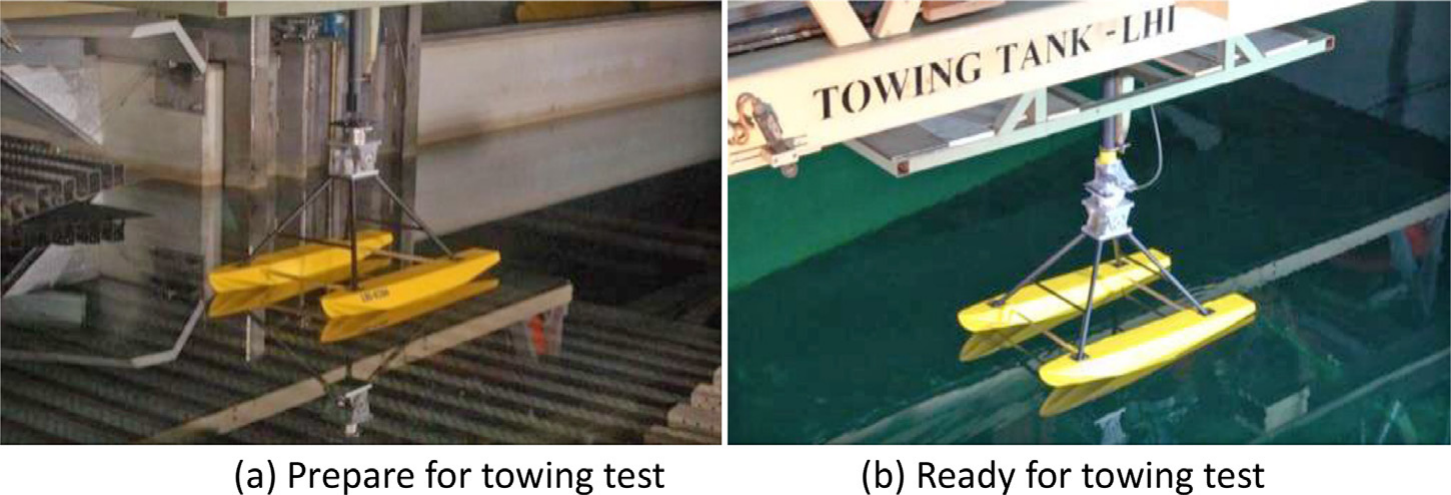
The Catamaran floater model scale 1:10 in towing tank Indonesian hydrodynamics laboratory.

The data collection presented consist of three folders, namely design of floater and cremona, measurement data, and video in towing tank. In the design of floater and cremona folder comprises 4 dwg and 3 jpeg files that display the sequence activities preparation of the hydrodynamic test. These include the geometry similarity of a model, floater, cremona, fixed moment reference point (fix-MRP), the catamaran and towing tank designs in Table 1, Figs. 1, 2, 3, 4, and 5, respectively. Then in folder measurement data consist of raw data for force and moment measurement dataset in towing tank (Indonesian Hydrodynamic Laboratory - BRIN) comprises 12 xls. These files demonstrate the sequence of activities for the hydrodynamic test, such as testing plan, towing tank, summary of data loadcell, force (F_x_, F_y_, and F_z_) and moment (M_x_, M_y_, M_z_) graphs as shown in Table 5, Figs. 10 and 11. The last folder was the towing tank video which contains test videos with various configurations which aim to find out the phenomena that occurred during the test and the video results were captured into photo as seen in Fig. 7, Fig. 8, Fig. 9.

**Table 1:** Geometry similarity of the catamaran floater scale 1:10.

Dimension	Symbol	Floater	Model
Scale	–	1	10
Length over all	LOA	9.80 m	980 mm
Breadth	B	1.58 m	158.1 mm
Height	H	1.35 m	135.1 mm
Draught	T	0.727 m	72.3 mm
Displacement	Δ	7,039 kg	6.867 kg
Centre line distance between floater	S	4.7 m	470 mm
Scale factor	λ	√10	
Time	t	3.162 s	1 s
Velocity	V	3.162 m/s	1 m/s
Scale factor	λ	1.025 × 10^3^	
Mass	M	1,025 kg	1 kg

## 3 Experimental Design, Materials and Methods

### 3.1 Design of the catamaran floater and cremona

The geometric similarity settings for the catamaran floater model are outlined Table 1. The design of the catamaran floater [1], fixed moment reference point, cremona, and integrated design models were shown through the data lines plan in Fig. 1, Fig. 2, Fig. 3, Fig. 4.

### 3.2 Manufacturing and setting of the catamaran floater model, cremona and fixed moment reference point

The manufacturing process of the catamaran floater model scale 1:10 and cremona is shown in Fig. 5. For the catamaran floater model used in test, the construction involved the utilization of a frame of cross-sectional multiplex and bark from wood. Subsequently, the model was coated with fiberglass, caulked, and painted to enhance its durability and performance. Due to the need to adapt to the available testing facilities, the size of test model was adjusted and scaled down. The ratio of the current model size to test model scale was 1:10, ensuring compatibility with testing setup. The cremona material used for construction consisted of cylinder pipe steel, while fixed moment reference point was made using stainless steel.

### 3.3 Test conditions with fixed moment reference point

Towing tank facility utilized for the hydrodynamic testing has dimensions of 234.5 m in length (including harbor), 11 m in width, and a water depth of 5.5 m. Towing tank facility used for the hydrodynamic testing has length, width, water depth, and maximum speed of 234.5 m (including harbor), 11 m, 5.5 m, and 9 m/s. No digital filter was used in LabView signal processing. The data was read by transducer sensors (see in Table 2 and 3) installed in cremona. A load cell display changed due to the catamaran floater being exposed to water movement, and if it was seen at around the 0.02-volt value, the experiment could start.

**Table 2:** Transducer sensor information.

Brand	Part Number	Description
ATI Industrial Automation	9105-TIF-Gamma-IP65	Gamma DAQ F/T transducer with IP65 protection
	9105-C-L-PS-5	DAQ IP60/65/68 transduces cable, IP68 Lemo connector to power supply, 10 m
	9105-PS-1	DAQ Transducer power supply
	9105-C-PS-U-2	Cable from power supply or interface power supply box to DAQ card, unterminated, 2 m length

**Table 3:** Transducer accuracies Gamma IP65.

Calibration	F_x_	F_y_	F_z_	T_x_	T_y_	T_z_
SI-130-10	0.75 %	1.00 %	1.00 %	1.00 %	1.25 %	1.50 %

**Fig. 6:**
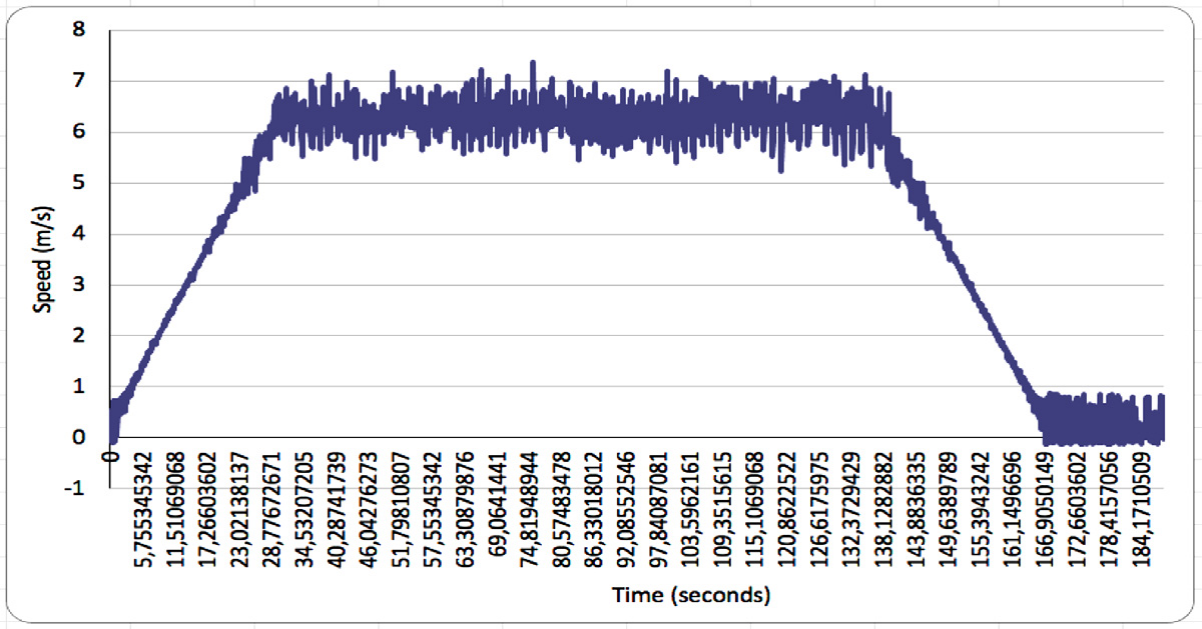
Time interval speed range 0 to 6 m/s in towing test.

Tests were carried out at various speed ranging from 0 to 6 m/s, with intervals of 1 m/s and used to collect hydrodynamic data for the catamaran floater. To achieve a speed range from 0 to 6 m/s in towing test, a time interval of 0 to 184 seconds was required as shown in Fig. 6. The catamaran floater design has a total resistance consisting of viscous and wave resistance. The viscous resistance is related to the Reynolds number, while wave resistance depends on the Froude [2,3]. Testing the resistance values aids in engineering designs for the catamaran floater, aimed to reduce water resistance, which predominantly involves considering floater's hull shape [4].

Additionally, the trim angle of floater varied from 0° to −2°, with intervals of 1°. The hydrodynamic testing plan in towing tank is shown in Table 4, specifying test speed and their corresponding actual speed. One vital factor contributing to water resistance is the trim angle, which significantly influences the ship's stability and hull durability [5,6].

**Table 4:** Testing conditions for the catamaran floater model.

No	ID	α = Trim_model_		V_model_	V_floater_	V_floater_
		°	Condition	m/s	m/s	knots
1	2309	0	even keel	1	3.171	6.164
2	2310	0	even keel	2	6.311	12.267
3	2311	0	even keel	3	9.454	18.378
4	2312	0	even keel	4	12.642	24.574
5	2301	-1	trim by stern	3	9.488	18.443
6	2302	-1	trim by stern	4	12.652	24.594
7	2303	−1	trim by stern	5	15.805	30.732
8	2304	−1	trim by stern	6	18.931	36.799
9	2305	−2	trim by stern	3	9.444	18.358
10	2306	−2	trim by stern	4	12.597	24.487
11	2307	−2	trim by stern	5	15.809	30.730
12	2308	−2	trim by stern	6	18.894	36.727

Test plan: no used ITTC 2014 standard, no repeat testing, and water temperature of 27°C

**Fig. 7:**
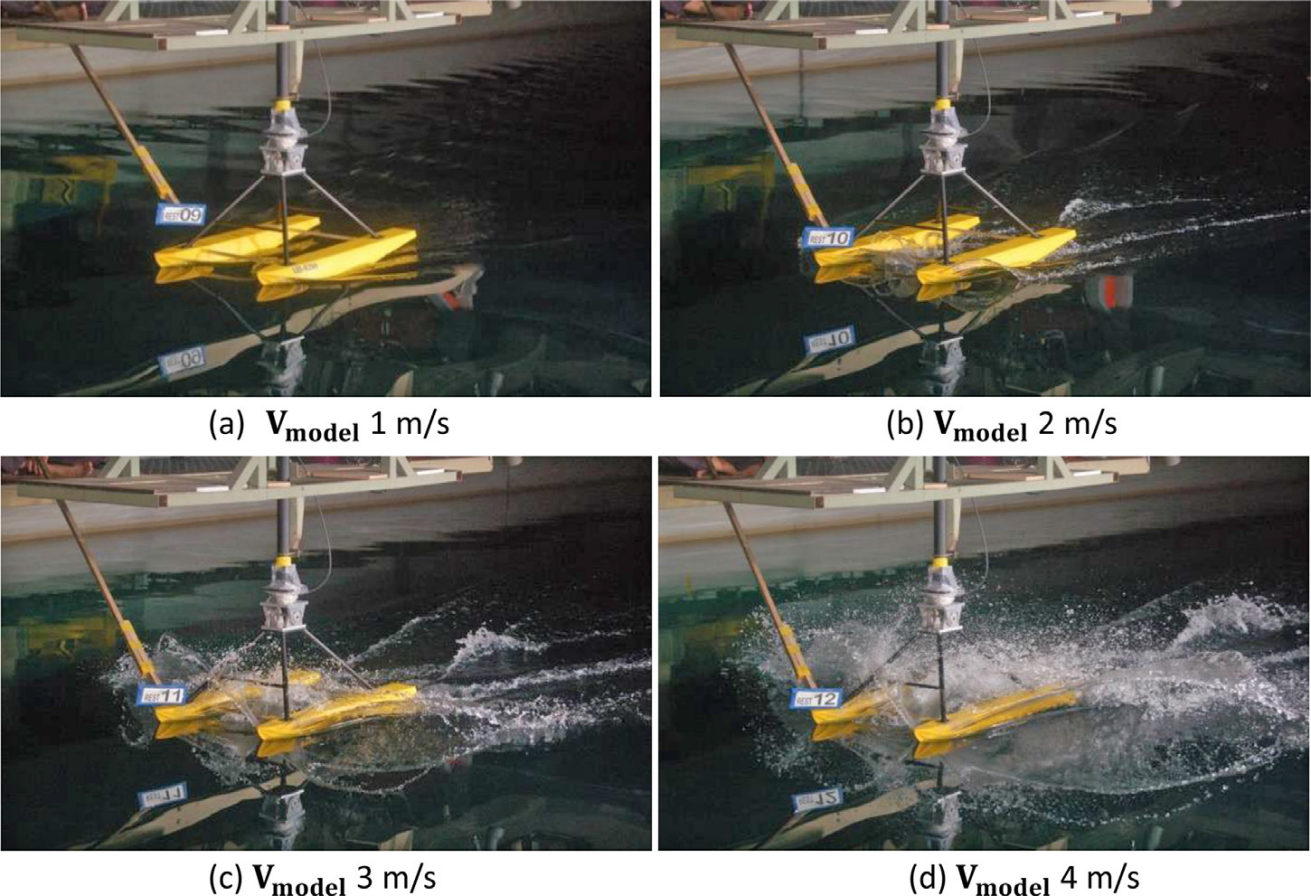
Flow pattern and water spray of the catamaran floater model scale 1:10 at trim 0°.

**Fig. 8:**
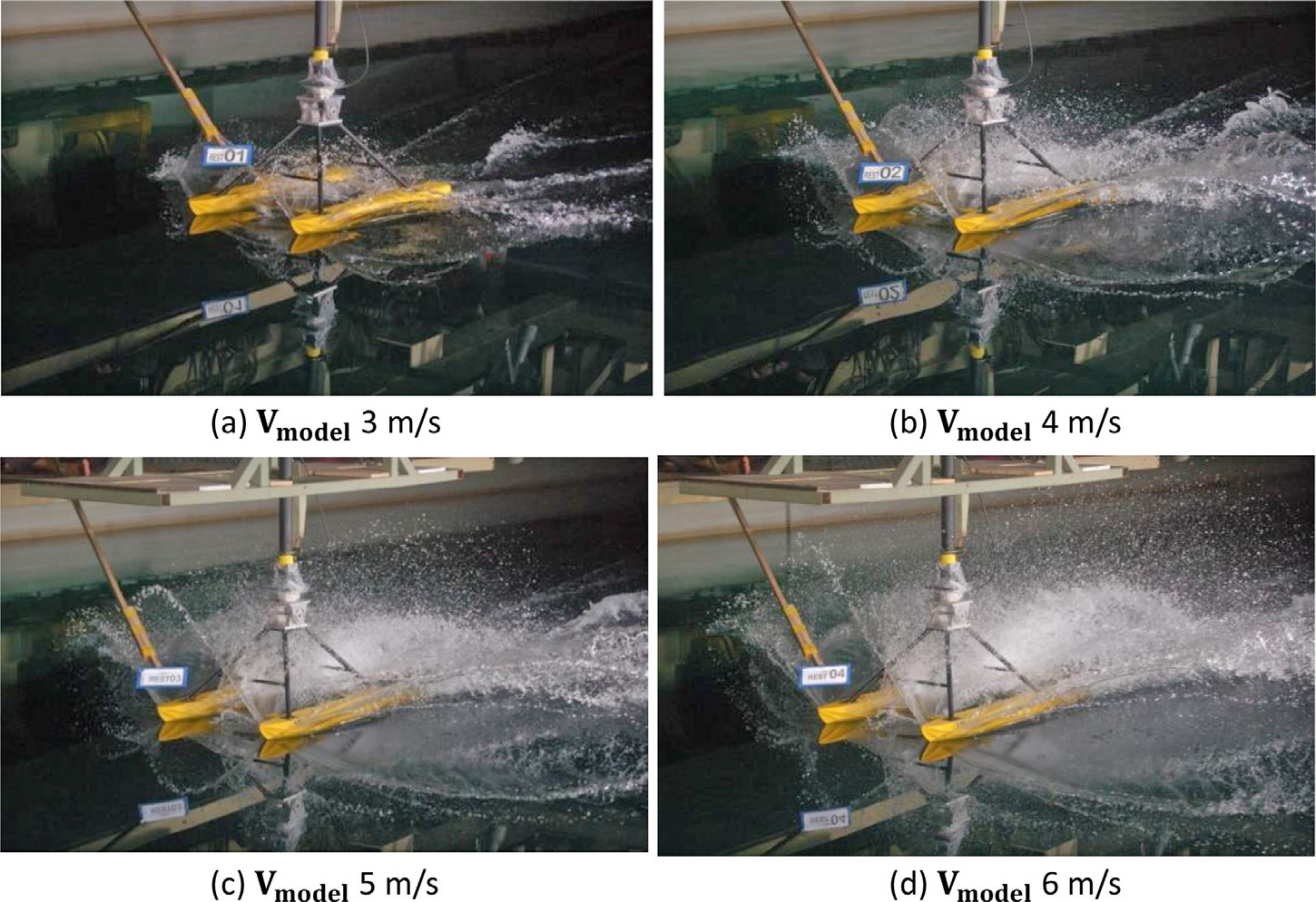
Flow pattern and water spray of the catamaran floater model scale 1:10 at trim −1°.

**Fig. 9:**
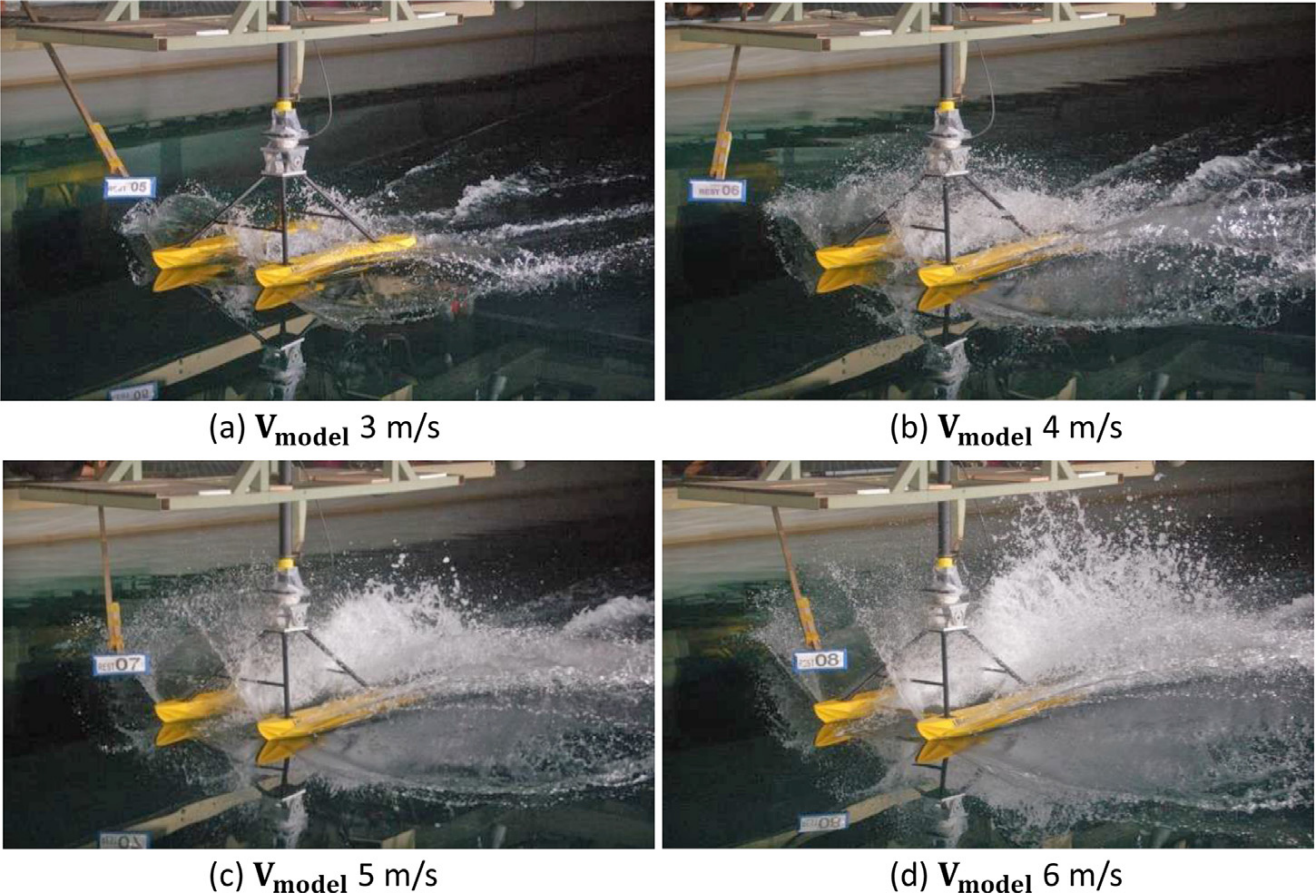
Flow pattern and water spray of the catamaran floater model scale 1:10 at trim −2°.

The visualization trim 0°, −1°, and −2° of the catamaran floater model scale 1:10 testing at towing tank are shown in Figs. 7, 8 and 9, respectively.

Fig. 7, Fig. 8, Fig. 9 show that the water spray phenomenon from the catamaran floater model experiences a displacement phase towards a planing hull with test speed of more than 3 m/s.

**Fig. 10:**
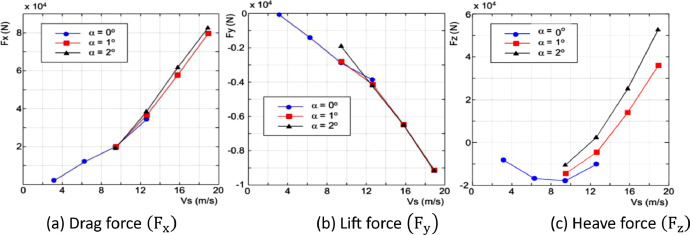
Graph of test result for force measurement.

**Fig. 11:**
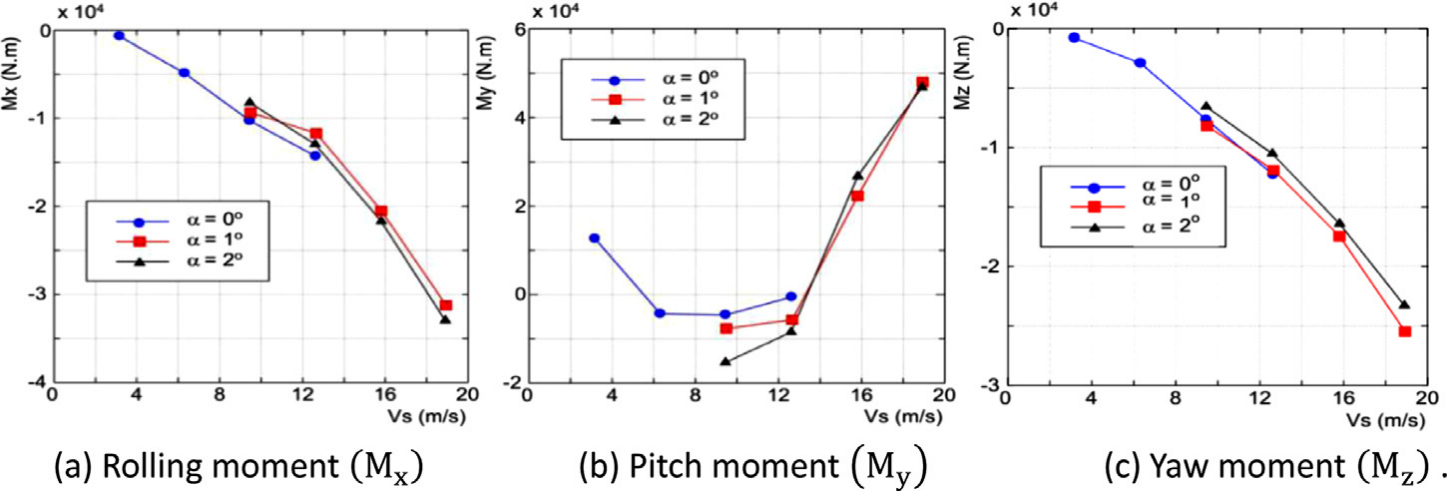
Graph of test result for moment measurement.

Test data output consists of drag force (F_x_), lift force (F_y_), heave force (F_z_), rolling moment (M_x_), pitch moment (M_y_), and yaw moment (M_z_) as shown in Table 5, Figs. 10 and 11. The uncertainty analysis of speed model as shown in Table 6.

**Table 5:** Testing data output of the catamaran floater model.

ID	Trim	V_model_	V_floater_	F_x_	F_y_	F_z_	M_x_	M_y_	M_z_
		m/s	m/s	N	N	N	Nm	Nm	Nm
2309	0°	1	3.171	2,033.678	−89.236	−8,563.295	−696.341	12,528.282	−809.933
2310	0°	2	6.311	12,027.550	−1,425.467	−16,836.443	−4,891.888	−4,360.570	−2,900.718
2311	0°	3	9.454	19,913.842	−2,890.095	−17,863.982	−10,272.524	−4,597.577	−7,659.967
2312	0°	4	12.642	34,331.890	−3,870.685	−10,161.921	−14,318.071	−562.691	−12,280.217
2301	−1°	3	9.488	19,895.333	−2,813.971	−14,43.802	−9,358.070	−7,672.130	−8,193.329
2302	−1°	4	12.652	36,533.911	−4,145.316	−4,519.035	−11,702.201	−5,691.708	−11,894.693
2303	−1°	5	15.805	57,665.078	−6,484.622	13,989.766	−20,512.635	22,268.004	−17,457.061
2304	−1°	6	18.931	79,630.751	−9,140.130	36,043.978	−31,236.973	48,008.238	−25,486.698
2305	−2°	3	9.444	19,014.268	−1,913.578	−10,570.430	−8,239.950	−15,361.766	−6,567.068
2306	−2°	4	12.597	38,28.926	−4,219.910	2,312.908	−12,955.040	−8,517.313	−10,510.383
2307	−2°	5	15.809	61,677.373	−6,513.687	25,082.156	−21,710.011	26,730.026	−16,401.545
2308	−2°	6	18.894	82,539.405	−9,168.575	52,600.088	−32,955.249	46,802.993	−23,285.330

**Table 6:** An uncertainty analysis for V_model._

ID	Trim	V_model_ (m/s)	Count no.	Average V_floater_	Standard Deviation	Min	Max
2309	0°	1	1,663	3.171	0.045	2.988	3.395
2310	0°	2	1,286	6.311	0.264	5.221	7.387
2311	0°	3	855	9.454	0.313	7.998	10.820
2312	0°	4	528	12.642	0.373	11.419	14.267
2301	−1°	3	1,075	9.488	0.306	8.233	10.906
2302	−1°	4	715	12.652	0.381	10.513	14.121
2303	−1°	5	591	15.805	0.366	14.431	16.912
2304	−1°	6	436	18.931	0.436	17.198	20.961
2305	−2°	3	824	9.44	0.334	8.144	11.076
2306	−2°	4	561	12.597	0.411	10.965	14.391
2307	−2°	5	459	15.809	0.428	14.096	18.039
2308	−2°	6	316	18.894	0.431	17.214	20.700

Figs. 10 and 11 illustrate a clear correlation between drag force (F_x_) and pitch moment (M_y_). The observed phenomenon from the catamaran floater, with a displacement phase towards a planning hull, showed an increasing drag force (F_x_) and pitch moment (M_x_) at test speed exceeds 9.4444 m/s.

Table 6 shows the uncertainty analysis on model speeds of 1 to 2 m/s having a standard deviation of less than 30 %, model speeds of 3 to 4 m/s having a standard deviation of 30 % to 40 %, and model speeds of 5 to 6 m/s having a standard deviation more than 40 %. The uncertainty analysis of trim intersection at V_model_ 3 m/s has a standard deviation of 0.306 to 0.334 (average 0.318), and V_model_ 4 m/s has a standard deviation of 0.373 to 0.411 (average 0.388).

## Limitations

Not applicable*.*

## Ethics Statement

The authors declare that the present work did not entail any experiments on human subjects, animals, and/or social media platforms.

## CRediT Author Statement

**Sayuti Syamsuar:** Conceptualization, Supervision, Writing – review; **Karyawan:** Design floater, Cremona and fixed moment reference point, Investigation; **Hendrato:** Design floater, Writing – original draft; **Yudiawan Fajar Kusuma:** Validation data, Writing – original draft and editing; **Muhammad:** Collecting data, Prepare model – review; **Sulistiya:** Collecting data, Prepare model – review; **Annissa Roschyntawati:** Collecting data, Prepare model – review; **Daif Rahuna:** Hydro test plan, Data acquisition and system; **Widyawasta:** Collecting data, Prepare cremona and fixed moment reference point – review; **Baharuddin Ali:** Manufacturing model – floater, cremona, fixed moment reference point, Towing tank activities; **Sutiyo:** Towing tank – review, Writing – review and editing; **Bagiyo Suwasono:** Conceptualization, Investigation, Methodology, Supervision, Writing – review and editing.

## Data Availability

Descriptive Floater98 with Fix-MRP in Towing Tank (Original data) (Mendeley Data).
